# The Ile191Val Variant of the TAS1R2 Subunit of Sweet Taste Receptors Is Associated With Reduced HbA_1c_ in a Human Cohort With Variable Levels of Glucose Homeostasis

**DOI:** 10.3389/fnut.2022.896205

**Published:** 2022-05-19

**Authors:** Joan Serrano, Fanchao Yi, Joshua Smith, Richard E. Pratley, George A. Kyriazis

**Affiliations:** ^1^Department of Biological Chemistry and Pharmacology, College of Medicine, The Ohio State University, Columbus, OH, United States; ^2^AdventHealth Translational Research Institute, Orlando, FL, United States

**Keywords:** HbA_1C_, *TAS1R2* gene, glucose homeostasis, sweet taste receptors, polymorphism, oral glucose tolerance test (OGTT), frequently sampled intravenous glucose tolerance test (FSIVGTT), diabetes risk

## Abstract

The Ile191Val variant of the *TAS1R2* gene of sweet taste receptors causes a partial loss-of-function and is associated with reduced glucose excursions in a healthy lean cohort. However, it is unclear whether this polymorphism contributes to the regulation of glucose homeostasis in metabolically unhealthy individuals. Thus, we used participants with variable glycemic profiles and obesity to assess the effects of the TAS1R2-Ile191Val variant. We found that the Val minor allele carriers had lower HbA_1c_ at all levels of fasting glucose and glucose tolerance. These effects were not due to differences in beta-cell function or insulin sensitivity assessed with a frequently sampled intravenous glucose tolerance test. This study extends our previous findings and provides further evidence that sweet taste receptor function may contribute to glucose regulation in humans.

## Introduction

Sweet taste receptors (STR; TAS1R2/TAS1R3 heterodimer) belong to the nutrient-sensing class of novel G-protein coupled receptors (GPCRs) that includes free fatty acid (i.e., GPR40,GPR120, CD36) and amino acid receptors (TAS1R1/TAS1R3; umami receptors). Among various tissues, these GPCRs are expressed in the gastrointestinal tract to integrate local and peripheral signals that modulate nutrient digestion and absorption (1). For instance, activation of STR can stimulate peptide release from mouse and human intestinal L-cells (2–4). In addition, STR promote glucose absorption in response to high concentrations of luminal glucose dependent on GLP-2 secretion and the apical translocation of GLUT2 transporter. Thus, genetic ablation of *Tas1r2* gene of STR in mice (TAS1R2-KO)reduces glucose absorption and its plasma excursions (5). Although there is some evidence that a similar mechanism may be present in humans (6, 7), the direct involvement of STR has not been clearly demonstrated. Interestingly, intestinal expression of STR in individuals with type 2 diabetes is linked to glucose absorption, suggesting that the levels and function of STR may contribute to postprandial hyperglycemia (8, 9).

We recently demonstrated that the *TAS1R2* (Ile191Val) polymorphism reduces the levels of STR in the plasma membrane, causing partial loss-of-function (10). Consequently, Val carriers had a mild reduction in glucose excursions in response to the ingestion of a glucose load, recapitulating the phenotype seen in TAS1R2-KO mice (5). Notably, the TAS1R2 (Ile191Val) variant is also associated with carbohydrate intake (11–13) and fasting insulin (11). Although these observations establish a strong link between STR function and glucose control, their clinical significance for the regulation of glycemia cannot be adequately assessed in metabolically healthy lean participants. To address this limitation and further explore the physiological manifestations of STR loss-of-function, we performed a retrospective observational study to assess the effects of Ile191Val polymorphism in a cohort of individuals with variable metabolic and obesity profiles.

## Methods

This prospective observational study was performed in accordance with the requirements of Good Clinical Practice and the Revised Declaration of Helsinki. Recruitment, enrollment and all study-related visits, including specimen collection and point-of-care laboratory testing, took place at Advent-Health Translational Research Institute (TRI) Clinical Research Unit (CRU), as previously described (NCT02226640) (14). The study was approved by the Institutional Review Board at Advent-Health and all participants signed an informed consent. All subjects were required to be at least 18 years of age, in general good health, with BMI <25 or > 30 kg/m^2^, but weight stable (<3 kg change within the last 8 weeks) and within 10% of their lifetime heaviest body weight. Non-diabetic participants were not taking medications known to affect glucose metabolism. Individuals with diabetes (HbA1c <8.0%) were either non-treated or were on monotherapy using either a sulfonylurea, metformin, or GLP-1 analog and were able to maintain accurate and reliable home glucose monitoring logs. Participants were excluded if one of the following conditions applied: Treatment with more than 2 of the following: metformin (Fortamet, Glucophage, Glumetza, Riomet), sulfonylureas (Glucotrol, Diabeta, Glynase, Micronase), Glucagon-like peptide-1 analogs (Byetta) and/or Dipeptidyl peptidase IV inhibitors (Januvia, Onglyza); Treatment with long acting Glucagon-like peptide-1 agonists within the last 3 months (i.e., exenatide once weekly), Treatment with thiazolidinediones (TZDs) (i.e. Avandia, Actos, Rezulin) within the last 3 months; Known, untreated thyroid disease or abnormal thyroid function blood test; Known diagnosis of liver disease (except NASH) or elevated liver function blood test; Known diagnosis of kidney disease or elevated kidney function blood test; Uncontrolled high blood pressure (BP > 140 systolic or > 90 diastolic); Start of or changes in oral contraceptives or hormone replacement therapy within the last 3 months; Use of drugs or alcohol (>3 drinks per day) within the last 5 years; Uncontrolled psychiatric disease that would interfere with study participation; History of cancer within the last 5 years (skin cancers, with the exception of melanoma, may be acceptable); History of organ transplant; History of heart attack within the last 6 months; Current treatment with blood thinners or antiplatelet medications that cannot be safely stopped for testing procedures; Current anemia; History of HIV, active Hepatitis B or C, or Tuberculosis; Presence of clinically significant abnormalities on electrocardiogram; Current smokers (smoking any nicotine or non-nicotine product within the past 3 months); Use of any medications known to influence glucose, fat and/or energy metabolism within the last 3 months (e.g., growth hormone therapy, glucocorticoids [steroids], etc.).

Participants were genotyped and retrospectively grouped according to rs35874116 (Ile191Val) or rs9701796 (Cys9Ser) TAS1R2 non-synonymous single nucleotide polymorphism (SNP). Mathematical modeling was performed as previously described for (a) beta-cell function, insulin sensitivity and insulin clearance (7), and (b) insulin sensitivity (Si) and the acute insulin response to glucose (AIRg) (15).

### Statistical Analysis

All data are represented as mean +/- standard error and plotted with Prism 9 (GraphPad Software). All participants were retrospectively assigned to two groups based on TAS1R2 genotypes. Statistical analyses were performed using jamovi 2.2.5 (jamovi team). Allele equilibrium, frequency, and SNP linkage were analyzed by Chi-square tests. Baseline characteristics and metabolic responses to the oral glucose tolerance test (OGTT) and frequently sampled intravenous glucose tolerance test (FSIVGTT) were analyzed with a general linear model approach using sex, age, BMI, fasting glucose, and 2 h glucose during the OGTT as covariates. Area under curve (AUC) glucose, insulin, and C-peptide were adjusted for baseline values. Non-parametric variables were log-transformed prior to analysis and all models were checked for multicolinearity and normal distribution of the residuals. Possible confounding effects were analyzed by introducing the variables of interest as covariates in a hierarchical model. Relationships between glycated hemoglobin (HbA_1c_) and other variables were analyzed as partial correlations after adjustment for sex, age and BMI.

### Data and Resource Availability

The datasets generated during and/or analyzed during the current study are available from the corresponding author upon reasonable request. No applicable resources were generated or analyzed during the current study.

## Results

**Figure 1:**
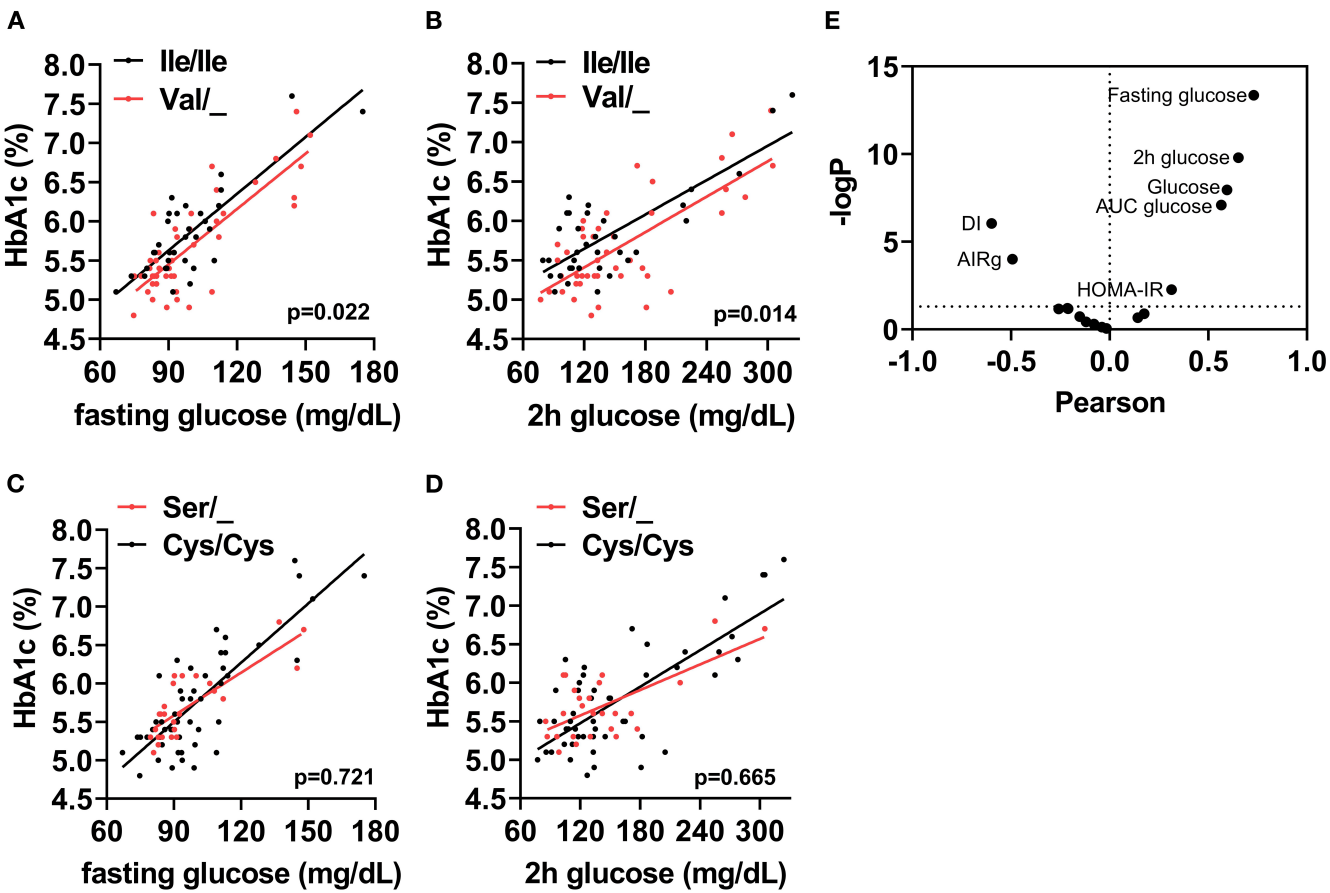
The *TAS1R2*-(Val) variant is associated with HbA_1c_ in humans. Association between **(A)** Fasting glucose, or **(B)** 2h glucose post OGTT (2h glucose) with percent glycated hemoglobin (HbA_1c_) in Ile/Ile (black) and Val carriers (red) using a linear regression model (p value for intercepts). **(C)** Fasting glucose, or **(D)** 2h glucose post OGTT (2h glucose) with percent glycated hemoglobin (HbA1c) in Cys/Cys (black) and Ser carriers (red) using a linear regression model (*p* value for intercepts). All values shown in A-D are unadjusted. **(E)** Correlation coefficient (Pearson) and statistical significance [–Log(p)] volcano plot for all assessed variables (i.e., baseline, OGTT and FSIVGTT) with HbA_1c_. Horizontal dotted line shows statistical significance of *p* <0.05 or higher. Only variables with *p* <0.05 are labeled. AUC, area under curve; DI, disposition index; AIRg, acute insulin response to glucose index; HOMA-IR, homeostatic model assessment for insulin resistance.

The cohort of participants had the expected Hardy-Weinberg equilibrium and minor allele frequency (Table 1). We specifically considered glucose tolerance along with gender, age, and BMI and performed multiple regression analysis between Ile/Ile and Val carriers (Val/_). We found that, at various levels of fasting glucose or glucose tolerance, Val carriers had lower HbA_1c_ (Table 2 and Figures 1A,B). The genotype effect on HbA_1c_ persisted even when the population was grouped according to their diabetes status (*p* = 0.040) based on American Diabetes Association (ADA) classification criteria (16) (i.e. normal glucose tolerance, pre- type 2 diabetes (T2D) and T2D) or when we only analyzed participants with normal fasting glucose and glucose tolerance (Ile/Ile 5.55 ± 0.07 vs Val/_ 5.34 ± 0.08, *p* = 0.046). Val/Val participants trended to have lower HbA_1c_, but the number of participants (total n=6) was inadequate to demonstrate statistical differences (Supplementary Table 1). Nevertheless, even after omitting Val/Val participants from the analysis, the HbA_1c_ differences between Ile/Ile and Val/Ile genotypes persisted (5.83 ± 0.05 vs 5.65 ± 0.05, respectively; *p* = 0.011). This suggests that heterozygosity is sufficient for the SNP effect on HbA_1c_. In contrast, the rs9701796 (Ser9Cys) polymorphism of *TAS1R2*, which has comparable allele frequency to Ile191Val (17), had no associations with HbA_1c_ (Table 2 and Figures 1C,D). No genotype differences were noted in OGTT variables or in insulin sensitivity or pancreatic beta-cell responsiveness during a FSIVGTT (Table 2).

**Table 1:** Allele frequency, distribution and linkage of participants.

	**Frequency**	**χ^2^**	***P***		**Frequency**	**χ^2^**	***P***
***Hardy Weinberg***				***Hardy Weinberg***			
**Ile/Ile**	35 (44%)			**Cys/Cys**	51 (65%)		
**Ile/Val**	39 (49%)	0.638	0.727	**Ser/Cys**	25 (32%)	0.000	>0.999
**Val/Val**	6 (8%)			**Ser/Ser**	3 (4%)		
***Allele distribution***				***Allele distribution***			
^*^Recorded (*n* = 216,414)				^*^Recorded (*n* = 114,744)			
**Ile**	68%	0.000	>0.999	**Cys**	78%	0.121	0.728
**Val**	32%			**Ser**	22%		
Observed (*n* = 80)				Observed (*n* = 79)			
**Ile**	68%			**Cys**	80%		
**Val**	32%			**Ser**	20%		
***Linkage between SNPs***		0.820	0.662				

*P values for Hardy-Weinberg equilibrium and allele distribution were obtained using chi square test. Linkage was obtained using chi square for association*.

* *L. Phan, Y. Jin, H. Zhang, W. Qiang, E. Shekhtman, D. Shao, D. Revoe, R. Villamarin, E. Ivanchenko, M. Kimura, Z. Y. Wang, L. Hao, N. Sharopova, M. Bihan, A. Sturcke, M. Lee, N. Popova, W. Wu, C. Bastiani, M. Ward, J. B. Holmes, V. Lyoshin, K. Kaur, E. Moyer, M. Feolo, and B. L. Kattman. “ALFA: Allele Frequency Aggregator.” National Center for Biotechnology Information, U.S. National Library of Medicine, 10 Mar. 2020, www.ncbi.nlm.nih.gov/snp/docs/gsr/alfa/*.

**Table 2:** Baseline and metabolic responses to an OGTT and FSIVGTT in adults with various levels of BMI and glucose control grouped by two common *TAS1R2* polymorphisms.

	**Ile/Ile**	**Val/_**	***P***	**Ser/_**	**Cys/Cys**	***P***
*Baseline variables*						
Total (Male/Female), *n*	35 (12/23)	45 (17/28)		28 (10/8)	51 (19/32)	
Age (y)	43.23 ± 2.13	41.22 ± 1.81	*0.473*	43.50 ± 2.32	41.39 ± 1.75	*0.473*
Height (cm)	171.00 ± 1.14	169.00 ± 1.01	*0.074*	171.00 ± 1.32	169.00 ± 0.97	*0.497*
Weight (Kg)	93.40 ± 1.38	90.50 ± 1.22	*0.106*	92.00 ± 1.58	91.00 ± 1.17	*0.629*
BMI (kg/m^2^)	31.62 ± 1.45	31.31 ± 1.38	*0.879*	29.49 ± 1.38	32.29 ± 1.33	*0.180*
Glucose (mg/dL)	97.64 ± 3.26	100.06 ± 3.20	*0.604*	96.37 ± 3.59	100.40 ± 3.00	*0.408*
Insulin (μU/ml)^*^	5.09 ± 1.01	7.10 ± 0.90	*0.538*	6.12 ± 1.17	6.16 ± 0.87	*0.560*
HbA1c (%)	5.82 ± 0.06	5.64 ± 0.05	*0.012*	5.77 ± 0.07	5.70 ± 0.05	*0.388*
Triglycerides (mg/dL)^*^	121.00 ± 11.90	128.00 ± 10.50	*0.305*	134.00 ± 13.42	121.00 ± 9.94	*0.895*
HDL (mg/dL)	55.90 ± 2.67	52.10 ± 2.37	*0.283*	51.90 ± 3.04	55.10 ± 2.25	*0.395*
LDL (mg/dL)	113.00 ± 5.90	101.00 ± 5.14	*0.130*	105.00 ± 6.79	107.00 ± 4.92	*0.725*
LDL/HDL	2.11 ± 0.15	2.10 ± 0.13	*0.971*	2.13 ± 0.17	2.08 ± 0.13	*0.814*
*OGTT variables*						
Baseline glucose (mg/dL)^*^	98.50 ± 0.32	98.10 ± 0.29	*0.614*	98.50 ± 0.36	98.10 ± 0.27	*0.574*
2h glucose (mg/dL)^*^	142.99 ± 10.32	155.62 ± 9.09	*0.201*	139.43 ± 9.84	156.74 ± 9.06	*0.957*
Baseline insulin (μU/ml)^*^	5.12 ± 0.90	6.90 ± 0.80	*0.637*	5.91 ± 1.04	6.09 ± 0.77	*0.564*
2h insulin (μU/ml)^*^	57.40 ± 11.11	70.20 ± 9.88	*0.508*	60.70 ± 12.70	65.60 ± 9.40	*0.559*
AUC glucose (mg/dL^*^min^*^10^−3^)^*^	18.35 ± 0.34	18.49 ± 0.30	*0.613*	18.44 ± 0.37	18.51 ± 0.28	*0.707*
AUC insulin (μU/L^*^min^*^10^−3^)^*^	5.49 ± 1.08	7.48 ± 0.96	*0.388*	5.98 ± 1.22	6.69 ± 0.90	*0.712*
AUC C-peptide (pmol/L^*^min)	725.00 ± 60.40	729.00 ± 51.10	*0.962*	793.00 ± 72.00	701.00 ± 46.40	*0.281*
HOMA-IR^*^	1.32 ± 0.26	1.85 ± 0.23	*0.689*	1.58 ± 0.30	1.60 ± 0.22	*0.553*
HOMA-B^*^	54.90 ± 9.09	69.70 ± 8.08	*0.667*	61.60 ± 10.45	62.80 ± 7.74	*0.654*
QUICKI	0.409 ± 0.008	0.408 ± 0.007	*0.931*	0.405 ± 0.009	0.413 ± 0.007	*0.534*
Matsuda Index	11.40 ± 1.06	11.00 ± 0.94	*0.778*	10.10 ± 1.19	11.90 ± 0.88	*0.226*
*FSIVGTT modeling analysis*						
S_I_(mL/kg/min/μU/mL)	4.40 ± 0.51	3.96 ± 0.41	*0.512*	3.80 ± 0.56	4.38 ± 0.40	*0.412*
S_G_ (mL/kg/min^*^10^3^)	13.62 ± 1.06	13.92 ± 0.84	*0.827*	14.34 ± 1.10	13.33 ± 0.78	*0.465*
AIR_G_ (μU/mL)^*^	391.67 ± 109.07	437.50 ± 86.00	*0.962*	415.93 ± 100.29	369.74 ± 71.10	*0.744*
Disposition Index (DI)	974.69 ± 130.02	912.11 ± 102.52	*0.711*	896.95 ± 135.98	924.96 ± 96.41	*0.869*

*All values are mean ± SEM. P value for genotype effect was obtained after adjustments for sex, age, BMI, fasting glucose, and 2h glucose using a general linear model. BMI, body mass index; HbA1c, glycated hemoglobin A1c. HDL, high density lipoproteins; LDL, low density lipoproteins; OGTT, oral glucose tolerance test; AUC, area under curve; HOMA-IR, Homeostatic Model Assessment for Insulin Resistance; HOMA-B, β-cell function; QUICKI, quantitative insulin-sensitivity check index; FSIVGTT, frequently sampled intravenous GTT; SI, insulin sensitivity index; SG, glucose effectiveness index; AIRG, acute insulin response to glucose index*.

* *Non-parametric data were log-transformed for statistical analyses*.

Therefore, we reasoned that HbA_1c_ levels might represent cumulative differences in other parameters related to glucose metabolism. Partial correlation analysis, adjusted for sex, age and BMI, demonstrated anticipated relationships between HbA_1c_ and basal, OGTT, or FSIVGTT parameters. Fasting and 2h glucose showed the strongest and most significant correlations with HbA_1c_ (Fasting: *r* = 0.75, p <0.001 and 2h: *r* = 0.65, *p* ≤ 0.001), suggesting that small changes in these variables, which are also descriptors of diabetes status, could explain cumulative differences in HbA_1c_. We also noted significant negative correlations with the disposition index (DI) and the AIRg of the FSIVGTT, and significant positive correlations with the homeostatic model assessment for insulin resistance (HOMA-IR), AUC glucose, fasting glucose and 2h glucose of the OGTT (Figure 1E). Similar correlations were found for both Ile/Ile and Val carriers (Supplementary Figures 1A,B). However, the addition of any of these parameters to our full regression model did not significantly decrease the standardized estimate for the SNP effect. Instead, the addition of DI further improved the regression model (Δ r^2^ = 0.04, model *p* = 0.014).

## Discussion

There is accumulating evidence to suggest that taste receptors, including STRs, regulate endocrine function (18). For instance, STRs regulate GLP-1 and GLP-2 secretion from intestinal L-cells to regulate incretin responses and glucose absorption (2, 5). In addition, STRs regulate insulin secretion directly on beta-cells in response to ingested sugars (19, 20) and artificial sweeteners (21, 22). These findings suggest that receptor-mediated “sweet” nutrient sensing is part of an intestinal-pancreatic axis that coordinates nutrient absorption and disposal.

These studies were primarily performed in cells and mice, so the direct involvement of STRs in human endocrine physiology is still ambiguous. This is primarily due to the absence of specific and potent pharmacological inhibitors or methods that directly assess STR function. Nevertheless, genomics approaches using SNPs has allowed scientists and clinicians alike to identify genetic markers that predict the present and development of a disease or screen for potential novel gene functions through various associations. STRs (TAS1R2/TAS1R3) are highly polymorphic (23), but *TAS1R2* in particular is characterized by high levels of nucleotide diversity (24). TAS1R2 also confers specificity to sweet taste, since TAS1R3 is involved in both sweet and umami taste (i.e. amino acid) (25). Out of the nine *TAS1R2* nonsynonymous SNPs, the rs35874116 (Ile191Val) and rs9701796 (Cys9Ser) have a minor allele frequency >0.2 and are associated with different nutritional and metabolic variables (17). *TAS1R2*-(Ile191Val) in particular is associated with sugar and carbohydrate consumption in adults (11, 13) and in children (12), but these effects are not due to differences in taste sensitivity (26). Taken together these observations suggest that, like in mice, TAS1R2 may have functional roles in peripheral tissues beyond taste perception. We recently used biochemical approaches to show that the Ile191Val substitution causes a partial loss-of-function of TAS1R2 by reducing the availability of the STR dimer in the plasma membrane (10). Healthy lean Val carriers had reduced glucose excursions during an OGTT (10), which resembles the effects seen in mice with a genetic loss-of-function of TAS1R2 (5, 27), confirming that the Val substitution causes a partial loss-of-function of STRs.

Because the rate of glucose excursions can affect the duration and magnitude of postprandial hyperglycemia (28), we explored contributions of *TAS1R2*-(Ile191Val) at baseline and during an OGTT or an FSIVGTT in a cohort of adults with various degrees of glucose control. We found that TAS1R2-(Val) carriers had reduced HbA_1c_, a measure that assesses progression of glycemic burden and predicts diabetic complications. The Ser9Cys substitution is located in the putative signal peptide of TAS1R2 and has been associated with dietary and anthropometric variables in children (17). However, the Ser9Cys variant did not affect HbA_1c_ levels or any other assessed variable. Although we cannot exclude the possibility of linkage disequilibrium with another causal polymorphism, the interactions of HbA_1c_ with the Ile191Val are not linked to Ser9Cys polymorphism. Unlike direct measures of fasting or postprandial plasma glucose, HbA_1c_ reflects mean glycaemia in the past 2–3 months, integrating total glucose exposure during fed and fasted states (29). Postprandial hyperglycemia significantly contributes to total daytime hyperglycemia and strongly correlates with HbA_1c_ (30). This finding is aligned with the reduced OGTT glucose excursions seen in metabolically healthy lean Val/_ participants (10). However, we did not observe a direct genotype effects in glucose or insulin responses during an OGTT. This may be partially explained by the population characteristics and the physiological factors affecting an OGTT. Previously, we used healthy lean adults with very homogeneous metabolic characteristics. This was deliberate in order to make phenotypic comparisons with corresponding healthy lean mouse models. Presently, the objective was to retrospectively assess the effects of TAS1R2 SNPs in a population with variable glucose status. This may have slightly reduced the power of our study considering that OGTT responses are not homogeneous across different levels of glucose intolerance and obesity status. This is likely due to the many factors that contribute to the development of glucose dysregulation (i.e., beta-cell function, insulin sensitivity, rate of glucose absorption) (31). Thus, interactions between these parameters can have differential effects on the OGTT responses.

To overcome this limitation, we reasoned that the HbA_1c_ differences might represent cumulative effects, so we set to identify which set of variables from the OGTT and FSIVGTT can account for the genotype association with HbA_1c_. The strongest correlations were noted with fasting and postprandial glucose (i.e., 2h post OGTT and AUC) along with indices of beta-cell function (i.e., AIR_G_ and DI). Although this is predictable, none of these variables reduced the regression coefficient of the model when added as covariate. Instead, adding DI as a covariate magnified the genotype effect. This suggests that the reduced HbA_1c_ in Val carriers could be mediated through amelioration of postprandial hyperglycemia linked to mechanisms that alter glucose absorption (32), instead of beta-cell function or insulin sensitivity. Although this is consistent with finding from animal models (5), to confirm this hypothesis in humans, clinical studies that directly measure glucose absorption are required. Notably, the genotype effect on HbA_1c_ persisted in normoglycemic participants, after exclusion of participants with abnormal glucose control (16). In addition, the Val allele is associated with lower consumption of sugars in obese (11), which could ameliorate the magnitude of postprandial hyperglycemia in this population. Therefore, although food intake was not recorded in this study, habitual differences in food choices and consumption may partially explain the lower HbA_1c._ Regardless of the associated mechanism, loss-of-function of STRs may predispose individuals to lower HbA_1c_ levels and confer a mild protective effect in daily glycaemia during the development of diabetes. This hypothesis should be confirmed through direct experimental evidence, such as in patients with continuous glucose monitors (33).

In conclusion, our studies highlight that, beyond taste perception, STR can act as peripheral carbohydrate sensors for the regulation of glucose homeostasis in humans. Particularly, partial loss-of-function of STRs through the *TAS1R2*-(Ile191Val) variant may confer beneficial effects in the regulation of daily glucose control. Our study was not adequately powered or designed to identify the mechanisms, but the genotype effects may be linked to differences in food preference and consumption, glucose excursions or other, yet unknown, peripheral mechanisms of glucose disposal. Notably, genome-wide association studies have yet to reveal independent contributions of *TAS1R2* polymorphisms on metabolic dysregulation, but careful consideration of appropriate covariates may be required to evaluate undelaying associations.

## Data Availability Statement

The raw data supporting the conclusions of this article will be made available by the authors, without undue reservation.

## Ethics Statement

The studies involving human participants were reviewed and approved by Institutional Review Board at Advent-Health, FL. The patients/participants provided their written informed consent to participate in this study.

## Author Contributions

JSe, RP, and GK designed experiments and interpreted data. JSe, JSm, and FY analyzed data. JSe and GK wrote manuscript. RP edited manuscript. GK conceived studies. All authors contributed to the article and approved the submitted version.

## Funding

This work was supported by the National Institute of Food and Agriculture (NIFA-2018-67001-28246 to GK), the National Institutes of Health (R01DK127444 to GK), the American Heart Association (AHA-904048 to JSe), and institutional support from the Ohio State University (to GK) and AdventHealth (to GK and RP).

## Conflict of Interest

The authors declare that the research was conducted in the absence of any commercial or financial relationships that could be construed as a potential conflict of interest.

## Publisher's Note

All claims expressed in this article are solely those of the authors and do not necessarily represent those of their affiliated organizations, or those of the publisher, the editors and the reviewers. Any product that may be evaluated in this article, or claim that may be made by its manufacturer, is not guaranteed or endorsed by the publisher.
