# Supplementary material for: The Ile191Val Variant of the TAS1R2 Subunit of Sweet Taste Receptors Is Associated With Reduced HbA1c in a Human Cohort With Variable Levels of Glucose Homeostasis
Source: Front Nutr. 2022 May 19;9:896205. doi: 10.3389/fnut.2022.896205 (PMC9160323; doi:10.3389/fnut.2022.896205)
Supplement: Supplementary file 1 [file Table_1.pdf]

Supp. Table.1

Supp. Table.1

HbA1c in participants with different Ile191Val zygosity.

	Genotype	HbA1c (%)	HbA1c (%) Adj. sex, age, BMI
**Normal glucose**	**Ile/Ile** (n=19)	5.59 ± 0.12	5.65 ± 0.11
	**Ile/Val** (n=17)	5.32 ± 0.13	5.51 ± 0.13
	**Val/Val** (n=3)	5.17 ± 0.30	5.36 ± 0.28
**Pre-T2DM and T2DM**	**Ile/Ile** (n=16)	6.08 ± 0.13	6.02 ± 0.12
	**Ile/Val** (n=22)	6.02 ± 0.11	5.90 ± 0.11
	**Val/Val** (n=3)	5.83 ± 0.30	5.92 ± 0.28

Adj., adjusted; T2DM, type2 diabetes mellitus
