# Supplementary material for: The Ile191Val Variant of the TAS1R2 Subunit of Sweet Taste Receptors Is Associated With Reduced HbA1c in a Human Cohort With Variable Levels of Glucose Homeostasis
Source: Front Nutr. 2022 May 19;9:896205. doi: 10.3389/fnut.2022.896205 (PMC9160323; doi:10.3389/fnut.2022.896205)
Supplement: Supplementary file 2 [file Image_1.pdf]

Supp. Figure.1

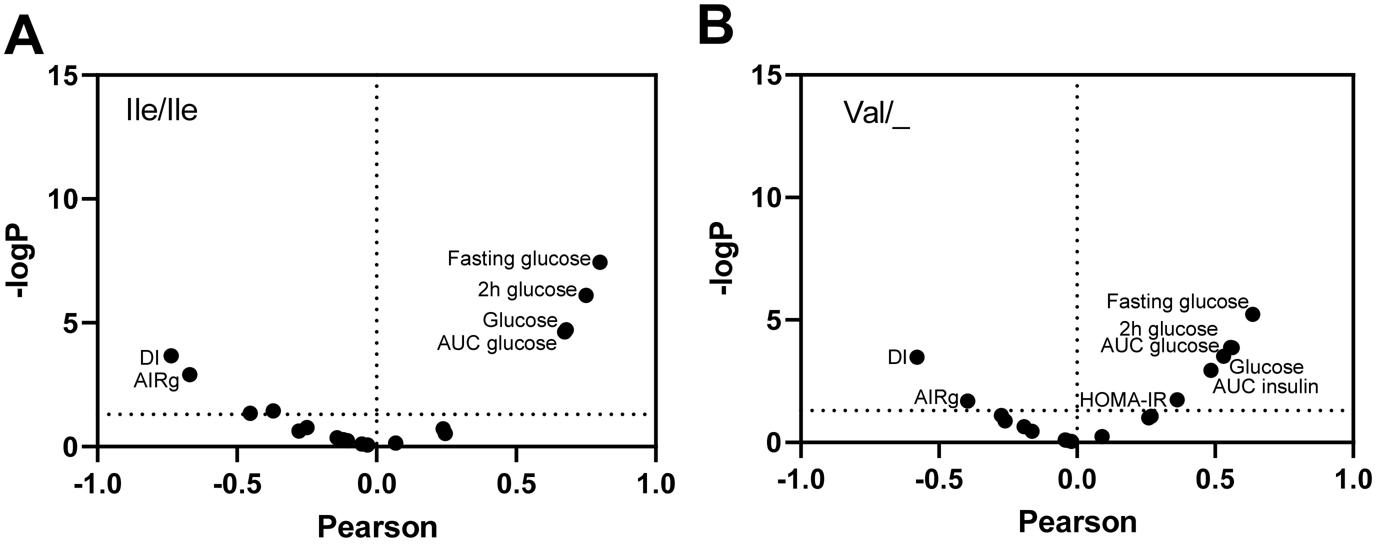

Correlation coefficient (Pearson) and statistical significance (-Log(p)) volcano plot in **(A)** Ile/Ile or **(B)** Val carriers for all assessed variables (i.e baseline, OGTT and FSIVGTT). Horizontal dotted line shows statistical significance of $p<0.05$ or higher. Only variables with $p<0.05$ are labeled. AUC, area under curve; DI, disposition index; AIRg, acute insulin response to glucose index; HOMA-IR, homeostatic model assessment for insulin resistance.
